# Chitin Nanocrystals Provide Antioxidant Activity to Polylactic Acid Films

**DOI:** 10.3390/polym14142965

**Published:** 2022-07-21

**Authors:** Murat Yanat, Ivanna Colijn, Karin Schroën

**Affiliations:** Laboratory of Food Process Engineering, Wageningen University and Research, Bornse Weilanden 9, 6708 WG Wageningen, The Netherlands; ivanna.colijn@wur.nl (I.C.); karin.schroen@wur.nl (K.S.)

**Keywords:** chitin nanocrystals, antioxidant activity, polylactic acid, nanocomposite, active packaging, DPPH

## Abstract

About 1/3rd of produced food goes to waste, and amongst others, advanced packaging concepts need to be developed to prevent this from happening. Here, we target the antioxidative functionality of food packaging to thus address food oxidation without the need for the addition of antioxidants to the food product, which is not desirable from a consumer point of view. Chitin nanocrystals (ChNC) have been shown to be promising bio-fillers for improving the mechanical strength of biodegradable plastics, but their potential as active components in plastic films is rather unexplored. In the current study, we investigate the antioxidant activity of chitin nanocrystals as such and as part of polylactic acid (PLA) films. This investigation was conducted using DPPH (1,1-diphenyl-2-picrylhydrazyl) radical scavenging activity. Chitin nanocrystals produced via acid hydrolysis showed five times higher activity compared to crude chitin powder. When using these crystals as part of a polylactic acid film (either inside or on top), in both scenarios, antioxidant activity was found, but the effect was considerably greater when the particles were at the surface of the film. This is an important proof of the principle that it is possible to create biodegradable plastics with additional functionality through the addition of ChNC.

## 1. Introduction

Oxidation is one of the greatest issues leading to food quality deterioration and, ultimately, food waste. This chain reaction is induced and propagated by radicals that react with atmospheric oxygen, thus directly affecting oxygen-sensitive food compounds, including proteins, vitamins, pigments, and especially unsaturated lipids [1,2,3,4]. Once initiated, the oxidation reaction will proceed, leading to food quality deterioration via nutrient and sensory losses such as off-odors, off-flavors, color or texture changes, and even the formation of toxic components. There are indications that products from oxidation reactions can react with biomolecules in the human body and thus impair human health due to cytotoxic, mutagenic, and carcinogenic effects [5,6]. As a consequence of all mentioned effects, a substantial reduction in food shelf life, and related to that, food loss, takes place [4]. From this, it is clear that it is crucial to reduce and ideally prevent food oxidation.

Conventionally, food oxidation is influenced by the addition of antioxidant compounds, for example, tocopherols, plant phenolics, or synthetic antioxidants such as butylated hydroxytoluene (BHT) and butylated hydroxyanisole (BHA). Although these methods can be very effective, there has been increasing (consumer) concern towards the addition of antioxidant compounds which are often presented as E-numbers on the food package. This is known to influence consumer acceptance and promotes so-called ‘clean-label strategies’ in food production. This makes it immediately clear that other strategies to prevent food oxidation are relevant and worthy of exploration. 

An alternative approach is the incorporation of bioactive compounds in the food package instead of in the food products themselves. Bioactive packaging has become quite a trend: in essence, a bioactive compound is used in food packages to maintain the quality and extend the shelf life of food [7,8,9]. The antioxidant action of bioactive films relies on the release of bioactive compounds such as organic acids, essential oils, fruit and plant extracts, etc., from the package to the food matrix. These antioxidants can be package-incorporated [10,11] or encapsulated by another substance [12,13,14] to achieve controlled release. Edible bioactive films are also available [15,16,17]. However, their applications are mostly limited to fruit and vegetable coating or short-term packaging. 

Considering the widespread use of bioactive films in food packaging, one of the main challenges is the heat sensitivity of the added antioxidant compounds. Commonly, high temperatures between 170–250 °C are used during plastic production, which results in the degradation of antioxidant compounds. For example, the degradation of essential oils can be as high as 90% during plastic extrusion [18]. Alternatively, it has been suggested to add small pouches containing antioxidant components after package material preparation, which in itself is a valid approach, but also comes with challenges (e.g., recycling of the pouches and recontamination of the food package through the pouch).

Heat-stable nanoparticles with antioxidative properties have been considered as part of the packaging material. In particular, metal nanoparticles such as silver, zinc oxide, copper, and titanium dioxide have been comprehensively studied in the literature [19,20,21]. They show great potential in terms of additional functionality, including antimicrobial and antioxidant activity, and UV-blocking properties, while simultaneously enhancing the material’s mechanical strength [20,22]. On the other hand, there are growing concerns about the migration of these nanoparticles to the food during storage, and also into the natural environment at the end of the lifetime of the package if not disposed of appropriately, or after incineration when not considered as part of appropriate waste management [23]. The impact on living organisms and natural ecosystems is potentially huge considering the non-biodegradable and long-lasting nature of these nanoparticles. This immediately makes it clear that biodegradable particles would be preferred, that is, if they can also supply the required functionality.

Others have shown the importance of enhancing circularity in food production and, more generally, in processing systems [24,25]. Creating high-value products by utilization of waste stream by-products is one of the key points. In the current paper, we develop nanocrystals from a biomaterial that is currently considered waste or, at most, a low-value by-product. In this way, we create not only added functionality but also added value. We do this using chitin, a linear copolymer consisting of 2-acetamido-2-deoxy-D-glucose linked together with β(1→4) glycosidic linkage. It is the second most abundant biopolymer in nature and is present in exoskeletons of crustaceans such as shrimps and crabs or cell walls of fungi [26]. Chitin powder and its derivative chitosan are well-known to scavenge free radicals in aqueous medium, thus illustrating their antioxidant activity [27]. The antioxidant action of chitin and chitosan is attributed to their hydroxyl and amino groups, which donate hydrogen to unstable free radicals [28,29] and thus terminate the radical chain reaction.

Chitin nanocrystals (ChNC) can be produced via the simple acid hydrolysis treatment of chitin powder and are known to improve the mechanical strength of biodegradable plastic (e.g., polylactic acid) and limit oxygen and water vapor transfer [30,31,32,33]. These beneficial attributes of nanocrystals are related to their large surface-to-volume ratio. In addition, in relation to the prevention of oxidative reactions, a high surface area is expected to be beneficial. In earlier work, it was already shown that the addition of ChNC to polylactic acid improved the mechanical strength of these bioplastics. These particles can also be used to create additional functionality in polymer films, which is rather unexplored, and here we take a major step toward using the particles to provide polymer films with antioxidant properties. 

In the present study, we focused on the antioxidant property of chitin nanocrystals as well as composite materials containing chitin nanocrystals and polylactic acid (the most prominent biobased plastic). This study differs from current bioactive packages in terms of antioxidative mechanisms that do not rely on an active ingredient transferring from the packaging material to the food matrix. We prepared chitin nanocrystals via acid hydrolysis and studied the nanocrystals as such or within nanocrystals/polylactic acid films using DPPH (1,1-diphenyl-2-picrylhydrazyl) radical scavenging. We studied the effect of particle size, ζ-potential, and degree of acetylation on antioxidant activity in the current manuscript. We demonstrated that the antioxidant activity of the nanocrystals and nanocomposite films is considerably higher compared to the initial chitin powder, which opens new routes to active packaging with additional functionality. 

## 2. Materials and Methods

### 2.1. Materials

Crude chitin powder (≥98% purity) was purchased from Glentham Life Sciences (Corsham, UK). Polylactic acid was obtained from Natureworks LLC (Ingeo Biopolymer 4043D, Plymouth, MN, USA). Calcofluor white stain (calcofluor white M2R 1 g/L and Evans blue 0.5 g/L) and all other chemicals or solvents were of analytical grade and supplied by Sigma-Aldrich (St. Louis, MO, USA). All dilutions were prepared with ultrapure Milli-Q water (Q-POD with Millipak Express 40 0.22 µm filter, MilliporeSigma, Burlington, MA, USA).

### 2.2. Sample Preparation

#### 2.2.1. Chitin Nanocrystals Preparation

ChNC were prepared by acid hydrolysis of crude chitin powder in 3 M hydrochloric acid (HCl) at 80 °C for 90 min under magnetic stirring. The hot mixture was cooled on ice to stop the reaction. The HCl was removed by centrifuging the samples at 4500× *g* for 5 min (Sorvall Lynx 4000, Thermo Fisher Scientific, Waltham, MA, USA), after which the supernatant was discarded, and the pellet was redispersed in an equal amount of MilliQ. This step was repeated three times. Subsequently, a 30 mL sample was added to a 50 mL reaction tube, and a sonification step was applied (5000 J energy input with an SFX150, Branson Ultrasonics, Brookfield, CT, USA) to increase chitin nanocrystal yield. Samples were diluted 10 times and centrifuged at 1200× *g* for 20 min. The supernatant containing the chitin nanocrystals was collected, and the pellet containing the amorphous chitin was discarded. A final centrifugation step was applied at 4500× *g* for 5 min to concentrate the chitin nanocrystal solution. The chitin nanocrystals were freeze dried before further usage (Christ Epsilon 2-6D, Martin Christ Gefriertrocknungsanlagen GmbH, Osterode am Harz, Germany). For this, samples were frozen at −40 °C for 12 h. The freeze-drying process was performed under a 1030 mbar vacuum. The temperature was increased from −20 °C to +20 °C in 8 stages during the freeze-drying process, which lasted for a total of 48 h.

#### 2.2.2. Nanocomposite Preparation

The solvent casting method, which is commonly used for the production of nanocomposites on a lab scale, was employed to produce PLA nanocomposites containing ChNC [34,35]. An amount of 5% (*w*/*v*) PLA chloroform solution was prepared and gently stirred for >12 h to ensure full solubilization. Likewise, the required amounts of nanocrystals were mixed in chloroform and vigorously stirred for 12 h. To create mixed matrix samples, PLA and ChNC solutions were mixed in desired amounts and left to stir for 3 h. The PLA/ChNC mixtures were poured into aluminum trays, and chloroform was allowed to evaporate in a fume hood. For ChNC positioned on top, first PLA−chloroform solution was poured into aluminum trays and left in the fume hood for 2 days to form a film. Afterwards, ChNC−chloroform suspension was added, which resulted in a ChNC layer on top of the PLA. All films were then placed in a vacuum oven at 40 °C for a week in order to remove chloroform fully.

#### 2.2.3. Deacetylation of ChNC

Alkaline treatment was used to increase the degree of deacetylation of ChNC [36]; ChNC was added to 50% NaOH (*w*/*v*) at 90 °C, and this mixture was continuously stirred for 1 h. After deacetylation, the samples were washed with Milli-Q water and centrifuged three times (4500× *g*, 5 min) to remove NaOH. The pH of the samples was adjusted to 5 using 0.1 M HCl. The deacetylated ChNC (D-ChNC) were freeze-dried using the same method described before for ChNC before further use.

### 2.3. Sample Characterization

#### 2.3.1. Particle Size and Zeta (ζ)-Potential

Laser diffraction (Mastersizer 3000, Malvern Instruments Ltd., Malvern, UK) and dynamic light scattering (Zetasizer Ultra, Malvern Instruments Ltd., Malvern, UK) were used to determine the size of chitin powder and chitin nanocrystals, respectively. The absorption index was set at 0.01, and refractive indices of 1.560, 1.360, and 1.330 were used for chitin, ethanol, and water, respectively.

The ζ-potential of all samples was measured through laser Doppler electrophoresis (Zetasizer Ultra). Size and ζ-potential analyses were conducted at 25 °C using capillary cells (DTS1070, Malvern Instruments Ltd., Malvern, UK). Prior to measurement, the pH of the sample was adjusted to 5 using 0.1 M HCl and 0.1 M NaOH.

#### 2.3.2. Viscosity Measurement

A stress-controlled rotational rheometer (MCR 301, Anton Paar, Austria) was used to investigate the behavior of ChNC in ethanol at various concentrations. A 50 mm cone plate (CP50-4, Anton Paar, Graz, Austria) was used in a shear sweep from 0.1 to 100 1/s.

#### 2.3.3. Degree of Deacetylation

A slightly modified version of the first derivative method described by Hein et al. (2008) [37] was used to determine the degree of deacetylation of samples. The reference substances, N-acetyl glucosamine (NAG) and D-glucosamine hydrochloride (GlcN.HCl), were dissolved in 85% (*w*/*w*) phosphoric acid to prepare 0.05 M reference solutions. Thereafter, the produced solutions were diluted with Milli-Q water to 1 mM, and a calibration curve was established (Appendix A, Figure A3). The powder and ChNC samples were dissolved in 85% (*w*/*w*) phosphoric acid under vigorous stirring at 55 °C for 60 min. After this, the sample solutions were diluted 40 times, and UV-VIS absorbance was measured at 210 nm (Beckman DU720, Beckman Coulter Inc., USA), with Milli-Q water as a blank solution. Experiments were carried out in triplicate, and values were averaged. Equation (1) was used to calculate the degree of deacetylation (DD, %).

(1) $$\mathrm{DD}=100\;\times \;\left(1\;-\;\frac{\mu \mathrm{mole}\;\mathrm{NAG}}{\mu \mathrm{mole}\;\mathrm{NAG}\;-\;\mu \mathrm{mole}\;\mathrm{GlcN}.\mathrm{HCl}}\right).$$

For µmole NAG, the calibration curve was used, and Equation (2) followed µmole GlcN.HCl, with *W* article weight (mg) per mL, and 0.203 and 0.161 conversion factors.

(2) $$\mu \mathrm{mole}\;\mathrm{GlcN}.\mathrm{HCl}=\frac{W\;-\;\left(\mu \mathrm{mole}\;\mathrm{NAG}\;\times \;0.203\right)}{0.161}.$$

#### 2.3.4. Nanocomposite Morphology

The distribution of ChNC in the polymer film was visualized using confocal laser scanning microscopy (CLSM) (SP5X-SMD, Leica Microsystems, Wetzlar, Germany) using calcofluor white for fluorescence labelling. ChNC and calcofluor white were mixed in dark conditions at room temperature for 30 min, after which 10% potassium hydroxide was added and left to stir for another 30 min. The mixture was diluted 10 times with Milli-Q water and centrifuged at 4000× *g* for 10 min, after which the supernatant was discarded, and the pellet was redispersed in an equal amount of Milli-Q water. This step was repeated three times. The particles were freeze-dried, after which they were ready for use, as explained before. 

For microscopy, nanocomposite samples were placed on an objective glass and attached by heating briefly. A 63x (water) objective (Leica Microsystems, Wetzlar, Germany) was used, in combination with a 480 nm filter, to observe the excited calcofluor white labelled-ChNC within the plastic matrix. Three hundred z-stack microscopic images were created per ~140 nm thick sample, and these images were processed with the software ImageJ (Fiji ImageJ 1.52, National Institutes of Health, Bethesda, MD, USA).

### 2.4. Antioxidant Activity

The antioxidant activity of ChNC and nanocomposites was determined following the DPPH (2,2-diphenyl-1-picrylhydrazyl) radical scavenging assay described by Brand-Williams et al. (1995) [38]. In short, a 0.5 mM DPPH/ethanol solution was prepared by stirring under dark conditions at room temperature until DPPH was completely dissolved. Next, particle/ethanol solutions were prepared by adding 375 mg particles to 25 mL ethanol. Approximately 0.5 mL of DPPH solution and 1.5 mL of particle solution were added to 2 mL tubes and stirred at 1400 rpm at 30 °C (Thermomixer C, Eppendorf AG, Hamburg, Germany) for 30 min. To prevent disturbance, samples were centrifuged for 5 min at 15,000 g (Eppendorf Centrifuge 5424, Eppendorf AG, Hamburg, Germany), after which the antioxidant activity of the supernatant was determined by measuring absorbance at 517 nm using a UV-VIS spectrophotometer (Beckman DU720, Beckman Coulter Inc., Brea, CA, USA), with ethanol as a blank and 0.125 mM DPPH in ethanol solution was a control. Each sample was measured in triplicate. Equation (3) was used to calculate radical scavenging activity in terms of DPPH inhibition, *I* (%).

(3) $$\;I\;\left(\%\right)=\left(\;\frac{Abs_{control}-Abs_{sample}}{Abs_{control}}\right)\times 100.$$

In which, *Abs_control_* and *Abs_sample_* indicate the absorbance values of DPPH + ethanol mixtures without and with the sample at 517 nm, respectively. To determine the antioxidant activity of nanocomposites, the films were immersed in DPPH/ethanol mixture for 8 h using the neat PLA film as control.

## 3. Results

### 3.1. Particle Characterization

Table 1 provide the size, ζ-potential, degree of deacetylation (DD%), and specific surface area of chitin nanocrystals (ChNC), deacetylated chitin nanocrystals (D-ChNC), and the starting chitin powder. Crude chitin powder possesses an average size of ~120 µm and a ζ-potential of +16.8 mV. Acid hydrolysis resulted in chitin nanocrystals (ChNC) with an average size of ~400 nm, which is a stark reduction compared to the starting material, as was also expected. Additionally, the harsh process conditions resulted in an increase in DD% of ~10% compared to the base material, and remarkably the ζ-potential almost doubled to +32 mV. Upon further alkaline treatment, the DD and ζ-potential increased strongly to 69% and sightly to +35.9 mV, respectively. These results are in line with the literature [39,40].

It is good to point out that the specific surface area of the ChNC may have been larger [41,42]. It is known that freeze drying results in strong aggregates that are difficult to redisperse and break up [43].

### 3.2. The Effect of Particle Size and Concentration on Radical Scavenging Activity

**Figure 1:**
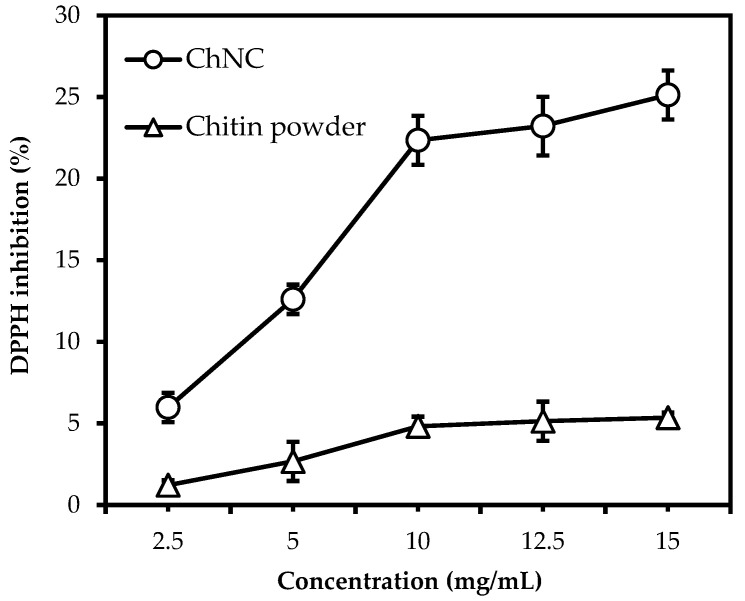
DPPH inhibition by ChNC (o) and chitin powder (Δ) for different concentrations.

The antioxidant activity was determined through DPPH radical scavenging assay; Figure 1 shows the DPPH inhibition (%) of crude chitin powder and ChNC at different concentrations after 30 min of incubation time in ethanol. Compared to crude chitin powder, the radical scavenging activity of ChNC was typically four to five times higher, and the activity did not increase completely proportionally with concentration. This may be caused by the difference in ratio between available surface area and substrate concentration, potentially going from a surface to a substrate-limited situation. Alternatively, crude chitin powder contains pores that can reach 250 nm [44,45]. This may lead to the underestimation of the specific surface area when measured by laser diffraction. Furthermore, the viscosity of the liquid may have influenced the reaction by slowing down the mass transfer. Especially the latter effect seems to be prominent in the ChNC systems that we investigated.

**Figure 2:**
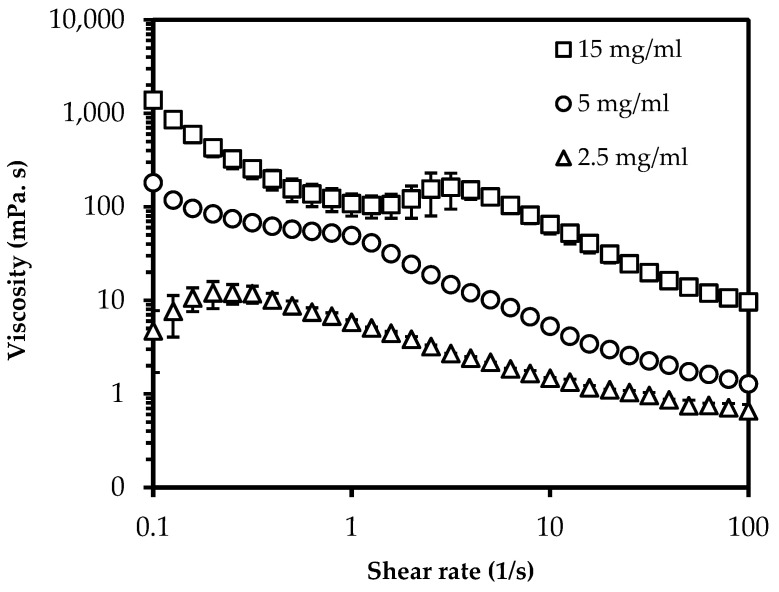
Viscosity of ChNC dispersions as function of shear rate.

**Figure A1:**
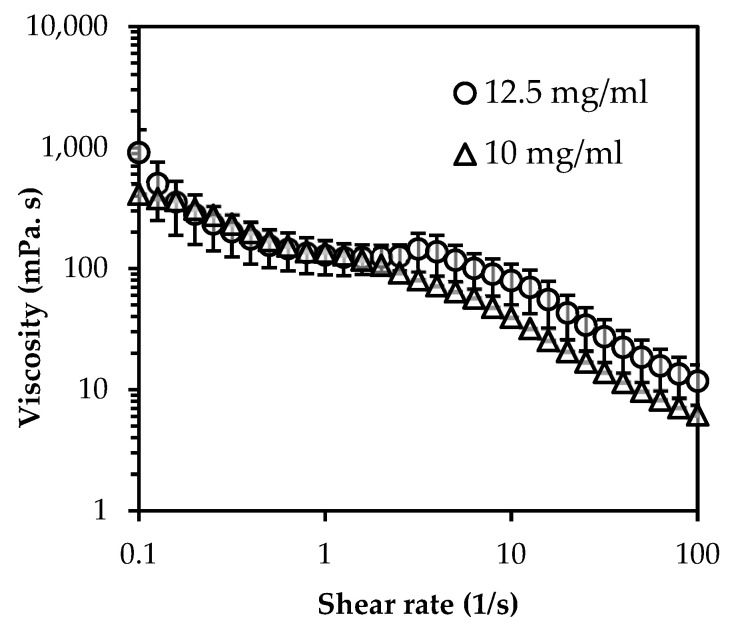
The viscosity of ChNC dispersions as function of shear rate.

Figure 2 shows the rheological behavior of ChNC/ethanol mixtures for shear rates between 0.1 1/s and 100 1/s. The viscosity of ChNC/ethanol suspensions drastically increases with ChNC concentration and shows complex behavior. This was most probably due to interparticle interactions, including hydrogen bonds and van der Waals interactions which result in a gel-like structure at high concentrations [46,47]. The viscosity at 0.1 1/s shear rate was 5, 181, and 1383 mPa.s for 2.5, 5.0, and 15.0 mg/mL particle concentrations, respectively. Moreover, the response at high particle concentration at shear rate region 1, −6 1/s, may be related to the disruption of the gel structure into sub-micrometer-sized elongated tactoids [48]. When tested, 10 mg/mL and 12.5 mg/mL ChNC/ethanol suspensions showed similar behavior (Appendix A, Figure A1). This gel-like structure is expected to result in limitations in the interaction between nanocrystals and the DPPH radical, leading to the levelling-off of antioxidant activity at concentrations above 10 mg/mL.

### 3.3. The Effect of pH and Degree of Deacetylation on Radical Scavenging Activity

**Figure 3:**
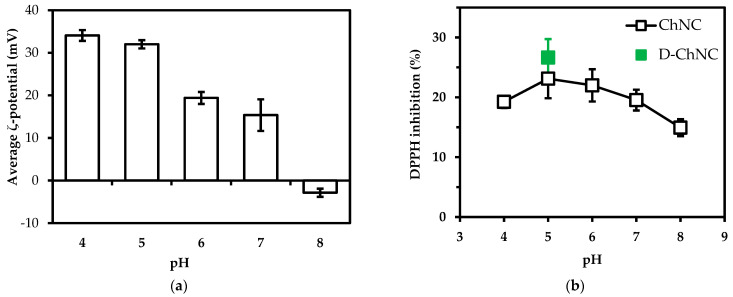
(**a**) The influence of pH on average ζ-potential of ChNC; (**b**) The DPPH inhibition (%) values at pH 4–8. 15 mg/mL concentration of ChNC was used during experiments.

The charge of the ChNC is expected to influence radical scavenging activity; therefore, the ζ-potential (Figure 3a) and DPPH inhibition were measured as a function of pH (Figure 3b). The average ζ-potential of ChNC was highest at pH 4 (+34 ± 1 mV). The high ζ-potential values at lower pH are the result of amino groups being highly protonated at acidic pH. At higher pH values, the average ζ-potential ultimately decreased to −3 ± 1 mV at pH 8.

**Figure A2:**
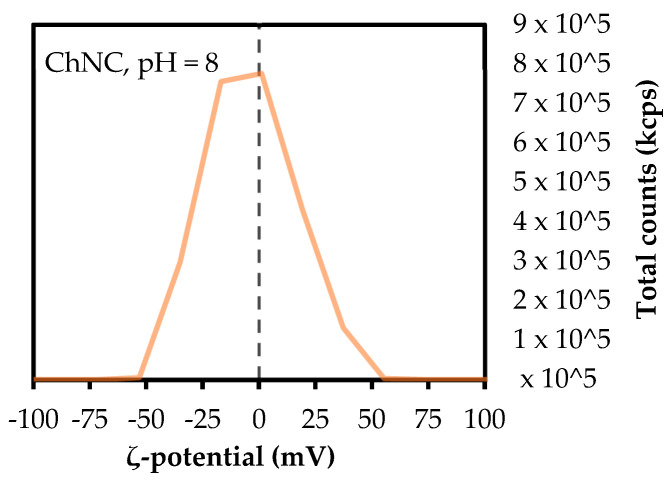
The ζ-potential (mV) distribution of ChNC at pH 8.

**Figure A3:**
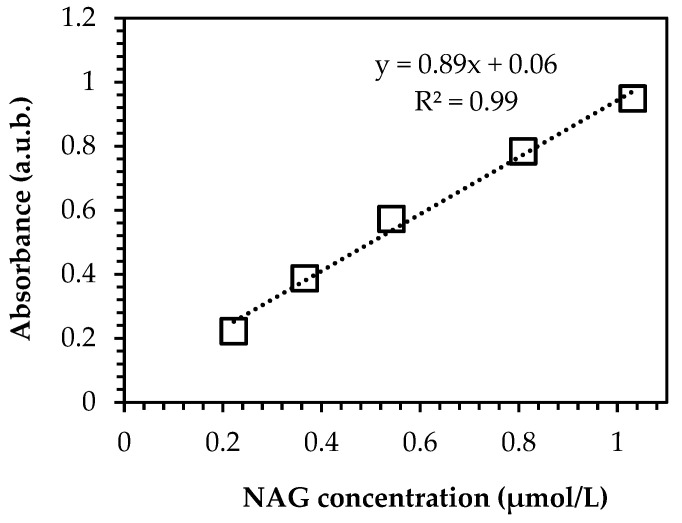
The calibration curve of NAG that was used to calculate the degree of acetylation.

Radical scavenging activity was much less pH dependent and remained over 15% for pH values 4–8 (pH-values as expected for foods (mostly < pH 7)). The radical scavenging activity for D-ChNC at pH 5 was similar to that for regular ChCN and for the ζ-potential (36 mV). In Figure 3b, we report on deacetylated chitin nanocrystals with ~70%DD, and it is good to share that at different DD (30–70%), we found no significant difference in radical scavenging activity (Appendix A, Table A1). It is good to point out that at pH 8, ChNC contains a distribution of charges; the distribution can be found in the Appendix A, Figure A2.

### 3.4. The Antioxidant Activity in Polylactic Acid Films

**Figure 4:**
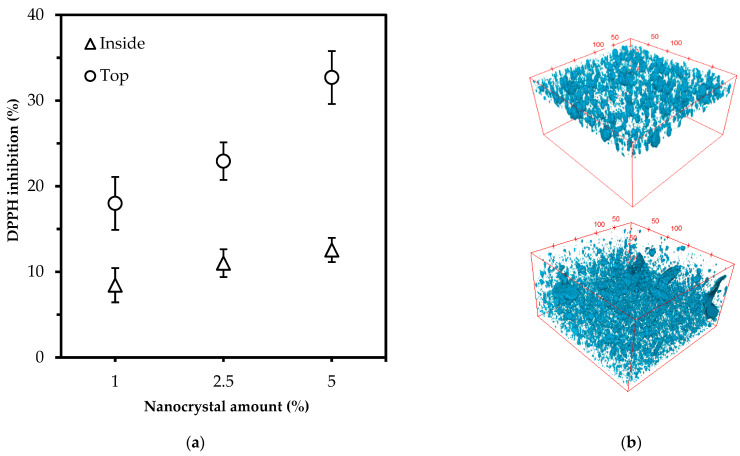
(**a**) DPPH inhibition (%) of ChNC/PLA nanocomposites after 8 h incubation time; (Δ) particles dispersed inside the plastic, (o) particles at the surface of the plastic; (**b**) CLSM micrographs of calcofluor white labelled ChNC in PLA (dimensions; 250 × 250 × 140 µm), and on top of PLA (1% on top, 5% inside the matrix, respectively).

ChNC were positioned inside and on top of polylactic acid films (Figure 4b), and radical scavenging activity was measured [38] at 1, 2.5, and 5% (*w*/*w*) (Figure 4a). The CLSM micrographs show that ChNC could be both distributed in PLA (bottom image) and positioned on top of a PLA film (top image). From Figure 4b, it is clear that chitin nanocrystal aggregation occurred during nanocomposite preparation, but it is good to point out that 90% of the particles were below 6 µm. When placed on top of the PLA film, the particles were found within a thickness of 35 µm.

The position of the ChNC clearly affected antioxidant activity. Overall, 1% ChNC placed on the surface showed higher activity (18%) than 5% ChNC dispersed inside the plastic (12.5%).Additionally, the radical scavenging activity increased much more strongly with particles deposited on top of the PLA film instead of inside. Still, both options can contribute to increasing the shelf life of food products through the proven radical scavenging effects.

## 4. Discussion

Protonation of amine groups (NH_3_^+^) has been related to antioxidant activity in chitin-derived products [49,50,51], and to date, most research is focused on chitosan, but our results indicate that chitin nanocrystals can be used as well. For example, we found DPPH scavenging activity of 22.5% at a concentration of 10 mg/mL ChNC after 30 min of incubation, and Yen et al. (2008) [52] reported 28.4% for chitosan at the same concentration and conditions, which is a comparable result. The ζ-potential of the particles is positive and more positive than the starting material, which will contribute to colloidal stability [53,54], as well as radical scavenging activity [55,56]. 

The antioxidant activity of ChNC is rather low compared to conventional antioxidants such as ascorbic acid, essential oils, tocopherols, or phenolics that commonly show a DPPH inhibition up to 75–90% at low concentrations (0.1–1 mg/mL) [57,58]. However, if these antioxidants were to be used as part of packing concepts, they would need to be resistant to the processing conditions used during the production of thermoplastic food packages. As these substances are sensitive to heat and high pressure [18,59,60], these conventional antioxidants will be degraded, mostly to a very great extent, during plastic production at ~170–250 °C [61] or need to be overdosed. In contrast, chitin nanocrystals are heat stable up to 250–300 °C [62,63] and, therefore, are better candidates for the production of active packages. When comparing chitosan with chitin nanoparticles, chitin has better miscibility than hydrophobic polylactic acid (chitosan is more hydrophilic) [64,65], which has been linked to improved mechanical and barrier properties [66,67,68,69].

For our envisioned application, active packaging, it is important that nanoparticles retain their antioxidant activity. Our results clearly show that ChNC possesses antioxidant activity, even when they are dispersed in the polylactic acid film, although they are more effective when positioned on top of the polymer film, which is both interesting leads for the design of active packages. Additionally, the activity occurred for the entire pH range investigated, meaning that ChNC can be used to prevent oxidation in pretty much any food ranging from animal-origin foods such as meat (raw), cheese (cottage, cheddar), and fermented dairy (yoghurt) to seafood (oysters, sardines, and tuna) [70]. These foods are commonly stored in plastic packages for retail sale. 

We expect that various approaches can be used to increase the antioxidant activity of ChNC further. In particular, the reduction of aggregation, which is expected to be possible through industrial-scale extrusion, can improve the dispersion of the particles in the plastic matrix [71,72], thus increasing the surface area available for antioxidative action. An alternative route is utilized to increase the antioxidant activity of ChNC by surface modification with a phenolic compound [73,74]; for instance, using Steglich esterification with caffeic acid [75]. This is expected to improve the dispersion and antioxidant activity simultaneously due to the hydrophobic nature of the added groups and their functionality. 

## 5. Conclusions

In this study, we produced chitin nanocrystals with an average size of 390 nm. These particles showed significant radical scavenging activity (DPPH assay) over a pH range from 4 to 8. When added to polylactide films, the chitin nanocrystals kept their antioxidant properties and transferred them to the polylactic acid film as a whole. The position of the nanocrystals in the film is an important design parameter, with particles on top of the package exhibiting significantly higher radical scavenging activity than those embedded in the film.

The findings in this paper will contribute to the development of active packaging concepts for biodegradable plastics that can be applied to different foods that require reduction of oxidation during storage and thereby contribute to extending shelf-life.

## Figures and Tables

**Table 1:** Particle characteristics of ChNC, D-ChNC, and chitin powder.

Sample	Size	ζ-Potential	Degree of Deacetylation	Specific Surface Area
	(μm)	(mV)	(%)	(m^2^/kg)
ChNC	0.4 ± 0.0	+32.0 ± 1.0	16.3 ± 1.6	285 ± 2
D-ChNC	0.8 ± 0.0	+35.9 ± 0.4	69.2 ± 3.8	184 ± 1
Chitin powder	122 ± 5	+16.8 ± 2.7	6.8 ± 1.1	35.2 ± 1

* Size measurements of ChNC and D-ChNC were carried out with Zetasizer Ultra, Mastersizer was used for chitin powder. ζ-potential values were measured at pH 5. For specific surface area measurement, samples were added to ethanol at 15 mg/mL.

## Data Availability

Data available on request.

## Appendix A

**Table A1:** DPPH inhibition (%) values of deacetylated chitin samples after 30 min of incubation with DPPH radical solution.

Sample	Degree of Deacetylation (%)	DPPH Inhibition (%)
D-ChNC	69.2 ± 3.8	26.63 ± 3.10
D2-ChNC	53.1 ± 5.2	22.7 ± 1.91
D3-ChNC	33.4 ± 2.8	20.8 ± 2.89
